# Multimodal computational neocortical anatomy in pediatric hippocampal sclerosis

**DOI:** 10.1002/acn3.634

**Published:** 2018-09-27

**Authors:** Sophie Adler, Mallory Blackwood, Gemma B. Northam, Roxana Gunny, Seok‐Jun Hong, Boris C. Bernhardt, Andrea Bernasconi, Neda Bernasconi, Thomas Jacques, Martin Tisdall, David W. Carmichael, J. Helen Cross, Torsten Baldeweg

**Affiliations:** ^1^ Developmental Neurosciences UCL Great Ormond Street Institute of Child Health University College London London United Kingdom; ^2^ Great Ormond Street Hospital for Children London United Kingdom; ^3^ Institute of Neurology University College London London United Kingdom; ^4^ Neuroimaging of Epilepsy Laboratory McConnell Brain Imaging Centre Montreal Neurological Institute and Hospital McGill University Montreal Quebec Canada; ^5^ Multimodal Imaging and Connectome Analysis Lab McConnell Brain Imaging Centre Montreal Neurological Institute McGill University Montreal Quebec Canada; ^6^ Developmental Biology and Cancer Programme UCL Great Ormond Street Institute of Child Health University College London London United Kingdom; ^7^ Department of Histopathology Great Ormond Street Hospital for Children NHS Foundation Trust London United Kingdom

## Abstract

**Objective:**

In contrast to adult cohorts, neocortical changes in epileptic children with hippocampal damage are not well characterized. Here, we mapped multimodal neocortical markers of epilepsy‐related structural compromise in a pediatric cohort of temporal lobe epilepsy and explored how they relate to clinical factors.

**Methods:**

We measured cortical thickness, gray–white matter intensity contrast and intracortical FLAIR intensity in 22 patients with hippocampal sclerosis (HS) and 30 controls. Surface‐based linear models assessed between‐group differences in morphological and MR signal intensity markers. Structural integrity of the hippocampus was measured by quantifying atrophy and FLAIR patterns. Linear models were used to evaluate the relationships between hippocampal and neocortical MRI markers and clinical factors.

**Results:**

In the hippocampus, patients demonstrated ipsilateral atrophy and bilateral FLAIR hyperintensity. In the neocortex, patients showed FLAIR signal hyperintensities and gray–white matter boundary blurring in the ipsilesional mesial and lateral temporal neocortex. In contrast, cortical thinning was minimal and restricted to a small area of the ipsilesional temporal pole. Furthermore, patients with a history of febrile convulsions demonstrated more pronounced FLAIR hyperintensity in the ipsilesional temporal neocortex.

**Interpretation:**

Pediatric HS patients do not yet demonstrate the widespread cortical thinning present in adult cohorts, which may reflect consequences of a protracted disease process. However, pronounced temporal neocortical FLAIR hyperintensity and blurring of the gray–white matter boundary are already detectable, suggesting that alterations in MR signal intensities may reflect a different underlying pathophysiology that is detectable earlier in the disease and more pervasive in patients with a history of febrile convulsions.

## Introduction

Hippocampal sclerosis (HS) is the most common pathology in adult epilepsy surgery series,^1^ and accounts for 15% of all resections in children. On histopathology, HS is characterized by segmental neuronal loss and gliosis within the hippocampal formation,^2^, ^3^ yet postmortem studies indicate neuronal loss and gliosis is also present in a proportion of patients in the amygdala,^4^ entorhinal cortex,^5^ temporal neocortex^6^ and extra‐temporal neocortex.^2^, ^7^

Neuroimaging provides the possibility to map these cytoarchitectural changes in vivo. Within the hippocampus, volumetric,^8^, ^9^, ^10^ T2 intensity,^11^, ^12^, ^13^ FLAIR intensity^14^ and advanced shape analyses^15^ have robustly quantified the pathological anomalies present in HS. In fact, correlational studies indicate the relationship of atrophy of the hippocampus with neuronal cell loss and T2 signal intensity with glial cell count, respectively.^11^, ^12^, ^13^

Beyond the hippocampus, there is a body of neuroimaging literature in adults, utilizing both volumetric and surface‐based methodologies, describing whole‐brain changes in gray matter volume,^16^ cortical thickness^17^, ^18^, ^19^ and T2/FLAIR intensity.^20^, ^21^ These changes are seen after several decades of illness duration, as seizures often start in childhood and adolescence. However, relative to the large number of studies mapping whole‐brain morphology and the distribution of T2 hyperintensity in adults with HS, relatively little is known about extra‐hippocampal neocortical brain changes in pediatric HS, except for small voxel‐based studies demonstrating focal reductions in gray matter density in medial and lateral temporal, as well as cingulate^22^ neocortices.^23^

In this study, using multimodal imaging to map whole‐brain changes both in the hippocampus and neocortex, we document epilepsy‐related brain pathology in pediatric HS. We then assess the relationship between these in vivo metrics and clinical factors, such as age of epilepsy onset, duration of epilepsy, history of febrile convulsions and postsurgical histology where available.

## Methods

### Participants

A retrospective cohort of patients with radiologically defined hippocampal sclerosis who underwent 3D T1‐weighted and FLAIR imaging on the 1.5T MRI scanner at Great Ormond Street Hospital (GOSH) as part of their clinical workup were studied, following permission by the hospital ethical review board. Cases were identified by searching the radiology reports between 2008 and 2015 for a radiological diagnosis of hippocampal sclerosis. Exclusion criteria were patients scanned using a different MRI scanner or protocol. Of the 34 patients identified who met these criteria, 12 further cases were excluded on the basis of poor MR scan quality. The following clinical information from the medical notes was gathered for the 22 remaining patients included in this study: age, sex, age at epilepsy onset, duration of epilepsy, radiological report, current anticonvulsant medications, history of febrile convulsions and, where applicable, postsurgical histology and Engel outcome. A control group of 30 term‐born children with no history of any neurological diagnosis were recruited by advertisement.

### Neurodevelopment/Cognition

In patients, verbal (VIQ) and performance (PIQ) IQ data from previous neuropsychological assessments performed at GOSH were collected. In patients younger than or equal to 7 years (*n* = 3), assessments used the Wechsler Preschool and Primary Scale of Intelligence (WPPSI). Whereas, the Wechsler Intelligence Scale for Children (WISC) was used in patients over 7 years old (*n* = 15). Cognitive data was available in 18/22 patients. All control participants were assessed using the Wechsler Abbreviated Scale of Intelligence (WASI) (*n* = 30).

### MR Imaging

All participants were scanned on a 1.5T Avanto MRI scanner (Siemens, Erlangen, Germany). Three‐dimensional data sets were acquired, using a T1‐weighted 3D‐FLASH sequence (TR = 11 msec, TE = 4.94 msec, FOV = 256 × 256 mm^2^, flip angle = 15°, voxel size = 1 × 1 × 1 mm^3^) and T2‐weighted FLAIR sequence (TR = 6000 msec, TE = 353 msec, TI = 2200 msec, FOV = 256 × 256 mm^2^, flip angle = 15°, voxel size = 1 × 1 × 1 mm^3^).

### Hippocampal segmentation

**Figure 1:**
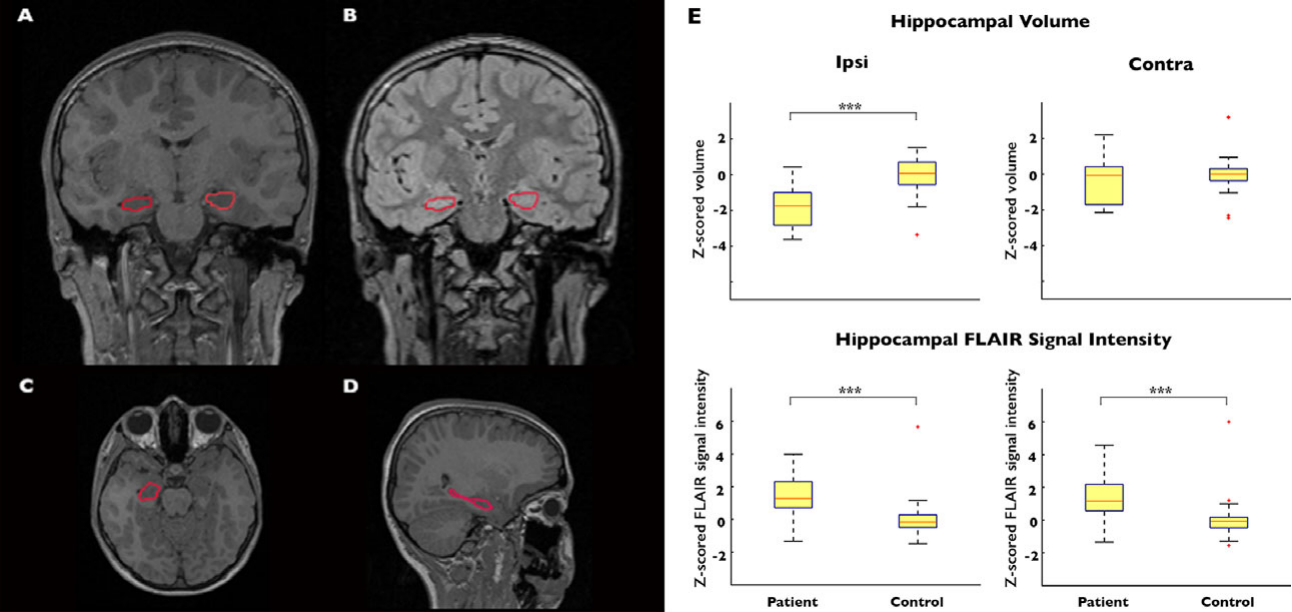
Hippocampal atrophy and FLAIR signal intensity. Hippocampal segmentations: coronal section of T1‐weighted (A) and FLAIR (B) images; and axial (C) and sagittal (D) T1‐weighted sections in a healthy control subject. E) Reduced ipsilateral but not contralateral hippocampal volume in patients. Increased normalized FLAIR signal of ipsilateral and contralateral hippocampi in patients.

Hippocampal segmentations were created, using FSL‐FIRST (v.5.0.8; http://fsl.fmrib.ox.ac.uk/). In brief, T1‐weighted images were initially registered to a standard template. FSL‐FIRST uses a Bayesian probabilistic model based on the shape and intensity information from a training set of 336 T1‐weighted MRI scans. FSL‐FIRST searches through linear combinations of shape modes of variation, from the training set, to find the most likely hippocampal shape given the T1 signal intensities from the image to be segmented.^24^ The segmentations were checked in Freeview and manually corrected in cases of overestimation into areas such as the lateral ventricle or underlying parahippocampal white matter (Fig. 1A–D). The reviewer was blind to the radiological report and confirmed side of lesion. Hippocampal volume and FLAIR signal intensity was calculated in FSL.

### Cortical reconstruction

**Figure 2:**
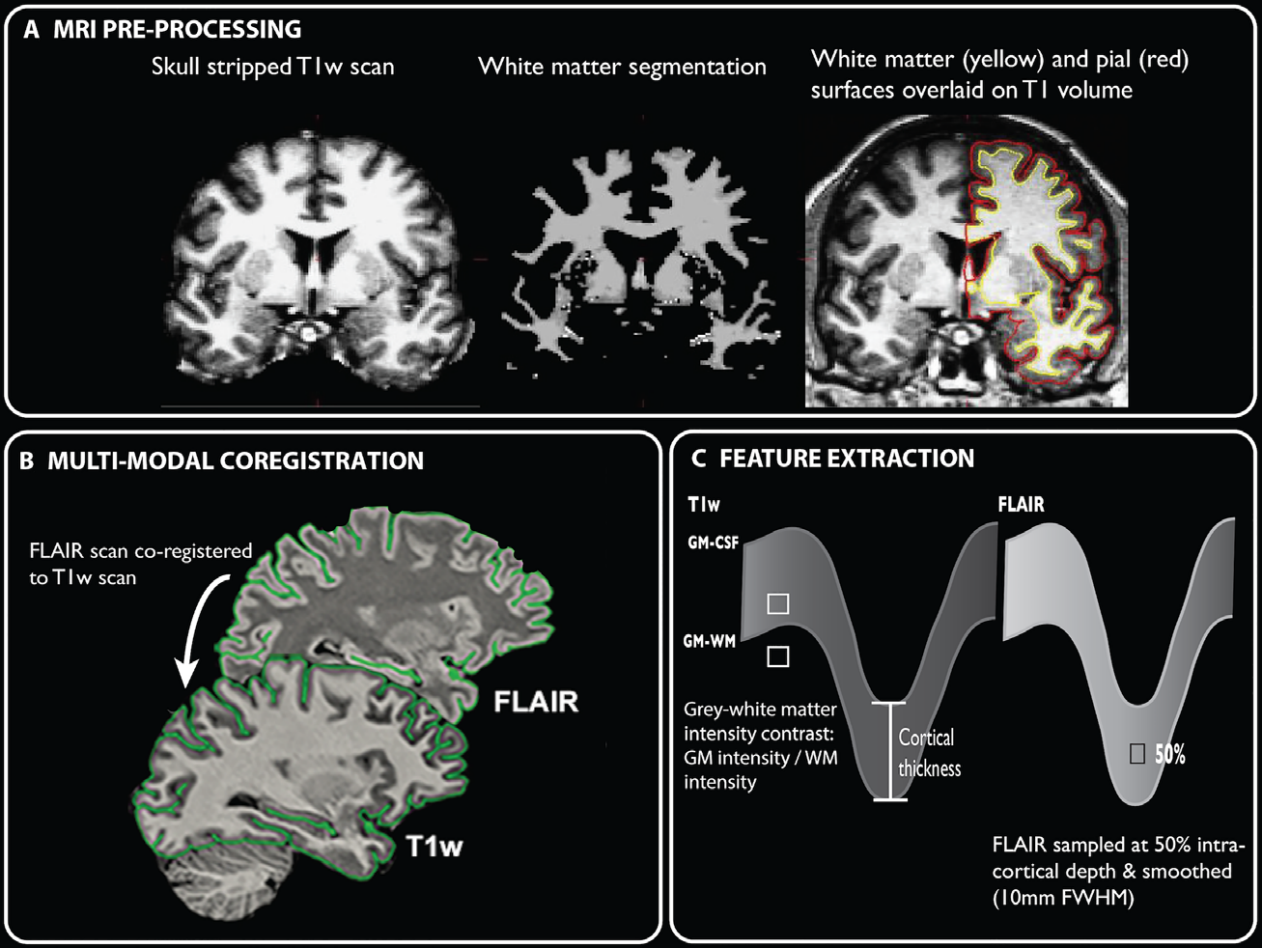
Surface‐based processing pipeline. (A) MRI preprocessing in FreeSurfer to create pial and white matter surfaces. (B) Co‐registration of FLAIR to T1 scans. (C) Feature extraction. Quantification of cortical thickness, gray–white matter boundary intensity contrast and FLAIR signal intensity at 50% cortical depth.

Cortical reconstructions were generated using *FreeSurfer* version 5.3.^25^, ^26^ MRI images were normalized for intensity and RF‐bias field inhomogeneities were removed, followed by skull‐stripping. Subsequently, images were classified into gray matter, white matter and cerebrospinal fluid. Hemispheres were separated, tessellated and deformed to create accurate smooth mesh representations of the gray–white matter interface and pial surface, with approximately 150,000 vertices per hemisphere (Fig. 2A). Within‐subject registration of FLAIR scans to T1‐weighted images was performed, using a boundary‐based cost function^25^ (Fig. 2B). All of the reconstructions were checked and any inaccuracies were manually corrected by M.B and verified by S.A.

### Neocortical morphological/intensity features

#### Cortical thickness

Cortical thickness was defined as the mean minimum distance between each vertex on the pial and white matter surfaces, generating a millimetre‐scale measure of the thickness of the cortex (Fig. 2C).

#### Gray–white matter intensity contrast

Gray–white matter intensity contrast was quantified because blurring of the gray–white matter boundary in the temporal pole is often visually identified in patients with HS. It was calculated on T1 images as the ratio of the absolute gray matter signal intensity to the absolute white matter signal intensity (Fig. 2C).^27^ The gray matter signal intensity was sampled at a distance of 30% of the cortical thickness above the gray–white matter boundary. The white matter signal intensity was sampled 1 mm below the gray–white matter boundary, using the *pctsurfcon* command in FreeSurfer. This has previously been demonstrated to be sensitive to blurring of the gray–white matter boundary in epilepsy lesions.^28^

#### FLAIR signal intensity

FLAIR signal intensity was sampled at 50% of the cortical thickness (Fig. 2C), to minimize partial volume effects, and normalized by the mode intensity of the GM/WM boundary across the brain. Normalization was necessary as FLAIR is not a quantitative MRI sequence and is not a ratio of intensities, unlike gray–white matter intensity contrast. In each participant, cortical per‐vertex and hippocampal FLAIR intensity normalization followed the equation:

RI(v)=(I(v) ‐ GMpeak)/(GMpeak ‐ B),

where *RI(v)* is the relative FLAIR intensity of a vertex, *I(v)* is the FLAIR signal intensity at this vertex, *GMpeak* is the mode of the FLAIR intensity histogram at 50% of the cortical thickness across the brain, and *B* is the mode of the intensity histogram at the gray–white matter boundary. This normalization procedure is adapted from a voxel‐wise method used in previously studies.^21^, ^29^

Cortical thickness, gray–white matter intensity contrast and normalized FLAIR intensity features were smoothed, using a 10 mm FWHM Gaussian diffusion kernel. Finally, individual normalized features were registered to an average space (fsaverage_sym) that has an identical number of vertices for each hemisphere.^30^

### Statistical analysis

Analyses were performed using SurfStat (http://www.math.mcgill.ca/keith/surfstat 31) for Matlab (MATLAB 6.1, The MathWorks Inc., Natick, MA, 2000). Patients were analyzed relative to the epileptogenic hemisphere (i.e., ipsi‐ and contra‐lesional to the seizure focus). However, before flipping, to control for regional variation of MRI feature, features were normalized using a between‐subject z‐scoring, where each participants per vertex feature was normalized by the mean and standard deviation in the population of healthy controls.

1. *Hippocampal volumetry and FLAIR signal intensity analysis* **:** Using SurfStat, linear models assessed between‐group differences in hippocampal volume and FLAIR signal intensity using age and intracranial volume as covariates of no interest. Within patients, paired *t*‐tests calculated differences between the affected and unaffected hippocampi.
2. *Mapping whole‐brain morphometric and intensity changes*: Vertex‐wise Student's *t*‐tests were used to compare cortical thickness, gray–white matter intensity contrast and normalized FLAIR signal intensity between patients and controls. All analyses controlled for age. Cohen's d effect sizes were calculated in clusters of findings. Surface‐based findings were corrected using random field theory for nonisotropic images,^32^ with a cluster‐defining threshold of 0.025 and controlling the family‐wise error (FWE) to be below P_FWE_ <0.05. This is necessary as when surface‐based data is projected onto a cortical surface, the different amounts of stretching of the surface alter the original constant FWHM used in the smoothing, making it nonisotropic.^32^
3. *Relation to clinical variables*: Correlation analyses and linear models were used to assess the effects of age of epilepsy onset, history of febrile convulsions and duration of epilepsy on MRI morphological and intensity features within the ipsi‐lesional hippocampus as well as within significant surface clusters established from *analysis 2*.

## Results

### Demographics

A total of 22 patients with a radiological diagnosis of HS (13 LTLE, mean ± SD age = 9.87 ± 3.37, range = 4.71–15.09 years, 7 females; 9 RTLE, mean ± SD age = 11.49 ± 3.18, range = 4.55–15.08 years, 6 females) and 30 healthy controls (mean age = 14.33 ± 3.01, range = 10.08–19.75 years, 13 females) were included. Demographic information for the patients is available in Table 1, and group characteristics in Table 2. 20 out of 22 had seizure semiology characteristic of TLE. The 2 remaining patients had nonlateralizing and unclear seizure semiology despite visible unilateral mesial temporal lobe sclerosis on MRI. As well as hippocampal sclerosis, radiological reports mentioned signal changes or blurring in the anterior temporal lobe in 10 out of 22 (45.45%).

**Table 1:** Pediatric HS patient demographics

	Age	Sex	Hemi	Onset	Duration	FC	VIQ	PIQ	Blurring	Surgery	Hippocampal histology	Temporal neocortical histology	Engel Outcome	AEDs
1	12.09	F	R	0.5	11.59	y	81	86	Y	Y	ILAE Type I HS	FCD IIIA	Ia	LVT, TPR
2	11.38	M	L	2	9.38	y	53	55	Y	N				TPR
3	13.75	F	L	4	9.75	n	108	125	N	Y	ILAE Type I HS	FCD IIIA	Ib	CBZ, LVT
4	7.03	F	L	4.5	2.53	n	112	110	N	N				LVT, VPA, OXCBZ
5	11.72	F	R	1.5	10.22	n	83	90	N	N				LVT
6	11.95	M	R	7	4.95	n	n.a.	n.a.	Y	N				None
7	13.57	F	R	0.83	12.74	y	87	86	N	N				VPA, OXCBZ
8	12.01	F	R	1.5	10.51	y	65	84	N	N				CBZ, TPR
9	8.68	F	L	1.5	7.18	y	87	88	N	Y	ILAE Type I HS	Nonspecific changes	Ia	LVT, VPA
10	13.92	F	R	0.92	13	y	108	100	Y	N				VPA
11	10.06	F	L	0.25	9.81	n	n.a.	n.a.	N	N				LVT, VPA
12	4.71	F	L	1	3.71	y	93	79	Y	Y	ILAE Type I HS	Nonspecific changes	IIIa	LVT, CLB, OXCBZ
13	8.53	M	R	1.5	7.03	y	69	77	Y	Y	ILAE Type I HS	Nonspecific changes	Ia	CBZ, LVT
14	8.01	F	L	1	7.01	y	99	94	Y	N				VPA, LVT
15	10.21	M	L	3.5	6.71	n	81	92	Y	Y	No diagnosis^a^	Nonspecific changes	Ia	TPR, LVT
16	14.87	M	L	9	5.87	n	105	109	N	Y	Gliosis only	Reactive cavities	IIa	LTG, CLB, zonisamide, perampanel
17	15.09	F	L	0.75	14.34	n	108	117	N	N				OXCBZ, LVT
18	4.55	F	R	0.75	3.8	n	n.a.	n.a.	Y	Y	ILAE type II HS^b^	Mild atrophy	Ia	VPA, LTG
19	6.95	M	L	0.33	6.62	n	45	49	Y	Y	ILAE Type I HS	Nonspecific changes	Ia	CBZ
20	11.63	M	L	7	4.63	n	98	100	N	N				VPA, TPR, LTG
21	5.96	M	L	5	0.96	n	n.a.	n.a.	N	N				Midazolam
22	15.08	M	R	12	3.08	n	n.a.	n.a.	N	N				LTG, VPA

Age, age of patient; Hemi, affected hippocampus; Onset, age of onset of epilepsy (years); Duration, duration of epilepsy (years); FC, history of febrile convulsions; VIQ, verbal IQ; PIQ, performance IQ; Blurring, radiological report of gray–white matter blurring in the anterior temporal lobe; Hippocampal histology: ILAE classification of hippocampal sclerosis; Ia, completely seizure free; Ib, nondisabling simple partial seizures only since surgery; IIa, Initially free of disabling seizures but has rare seizures now; III, worthwhile improvement; IV, no worthwhile improvement; AEDs, anti‐epileptic drugs at time of MRI; LVT, levetiracetam; CBZ, carbamazepine; OXCBZ, oxcarbazepine; VPA, sodium valproate; CLB, clobazam; LTG, lamotrigine, TPR, topiramate; PNT, phenytoin.

a No diagnosis as hippocampus not submitted.

b Inconclusive due to incomplete sampling of the hippocampus. Temporal neocortical histology: histopathological changes in samples temporal lobe neocortex. Engel: postoperative surgical outcome according to Engel classification.

**Table 2:** Group demographic data

	HS (*n* = 22)	CONTROLS (*n*=30)	*P*
AGE (mean + SD)	10.53 ± 3.32	14.33 ± 3.01	0.001
SEX (female:male)	13:9	13:17	0.17
VIQ (mean ± SD)	89.59 ± 17.83	113.13 ± 14.61	<0.001
PIQ (mean ± SD)	92.35 ± 19.57	115.73 ± 13.65	<0.001
ONSET (mean ± SD)	3.02 ± 3.20	n.a.	
DURATION (mean ± SD)	7.52 ± 3.69	n.a.	
HEMI (left:right)	13:9	n.a.	
SURGERY	45.5% (10/22)	n.a.	
FC	40.9% (9/22)	n.a.	

AGE, age of participants (years); VIQ, verbal IQ; PIQ, performance IQ; ONSET, age of onset of epilepsy (years); DURATION, duration of epilepsy (years); HEMI, affected hippocampus; SURGERY, patient has undergone temporal lobectomy; FC, history of febrile convulsions.

At the time of study, 9 out of 22 patients had undergone an anterior temporal lobectomy (Table 1). Histological analysis of the resected specimen revealed neuronal cell loss and gliosis in CA1 and CA4 subfields in 6 (International League Against Epilepsy (ILAE) HS type 1). In the remaining 3 operated patients, 1 had hippocampal sclerosis favoring ILAE HS type 2 (neuronal loss predominantly in CA1) but inconclusive due to incomplete sampling, 1 had gliosis only without detectable neuronal loss and the final patient had no diagnosis as the hippocampus was not submitted for histopathological diagnosis. 2 out 9 patients had FCD type IIIa, where there is temporal neocortical laminar abnormalities associated with HS. In 5 of the remaining 7 patients, nonspecific histological changes were identified in the temporal neocortex, such as subpial (Chaslin's) gliosis and white matter perivascular abnormalities. 1 patient had some reactive changes in the temporal neocortex thought to be due to the placement of depth electrodes and the remaining patient had some mild temporal neocortical atrophy but nil else. Among the 9 operated patients, 7 (78%) had Engel class I outcome, 1 (11%) had class II and 1 (11%) had class III outcome.

### Hippocampal changes

Between‐group quantitative analysis of hippocampal volume and normalized FLAIR intensity, with intracranial volume and age as covariates of no interest, revealed atrophy of the ipsi‐lesional (*t* = 3.21, *P* = 0.001; Fig. 1E) but not contra‐lesional (*t* = 0.87, *P* = 0.8; Fig. 1E) hippocampus and bilateral FLAIR hyperintensities (ipsi‐lesional: *t* = 3.86, *P* < 0.001; Fig. 1E; contra‐lesional: *t* = 3.35, *P* < 0.001; Fig. 1E) in HS patients compared to controls. Within patients, paired *t*‐tests indicated significantly more ipsi‐ compared to contra‐lesional hippocampal atrophy (*t* = 7.06, *P* < 0.001) and FLAIR hyperintensity (*t* = 2.32, *P* = 0.03). In pediatric HS patients, ipsi‐lesional hippocampal volume (mean ± SD) was 2780 ± 341 mm^3^ compared to 3210 ± 388 mm^3^ on the contra‐lesional side. In controls, hippocampal volume was 3310 ± 278 mm^3^ on the left and 3280 ± 312 mm^3^ on the right.

### Topography of neocortical changes

**Figure 3:**
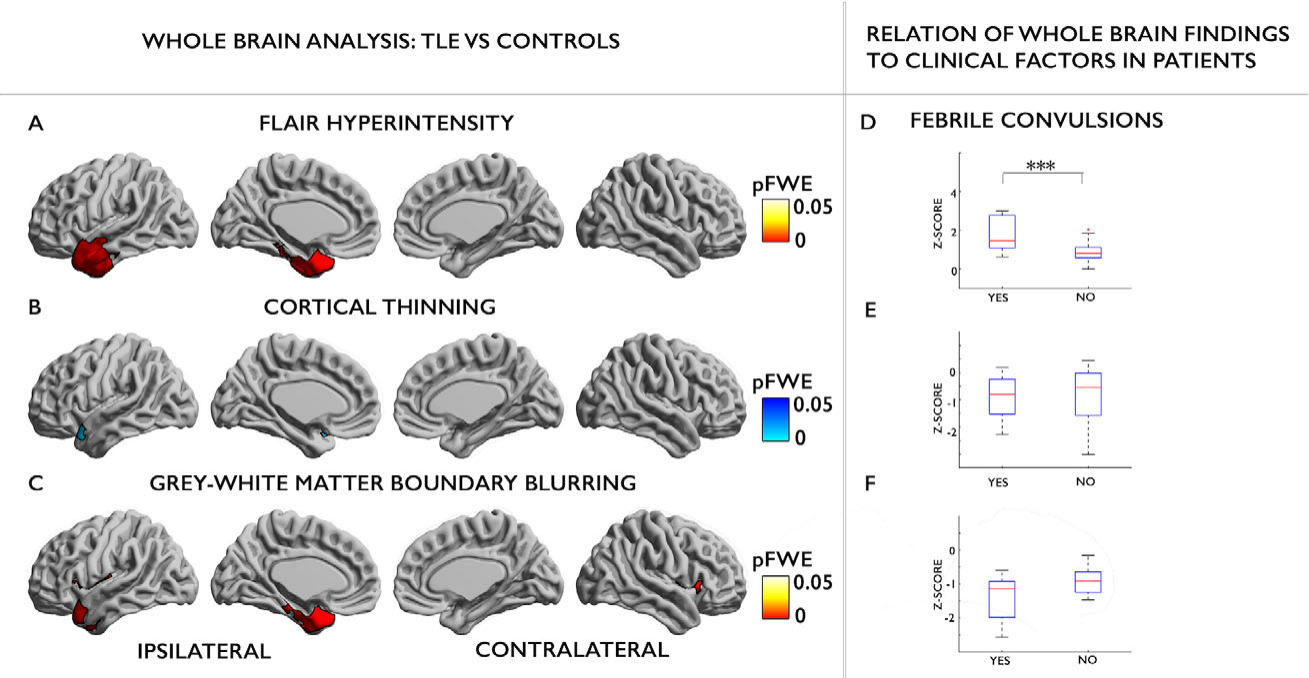
Whole‐brain analysis. FLAIR hyperintensity (A), cortical thinning (B) and gray–white matter boundary blurring (C) in pediatric TLE patients compared to controls. Findings were corrected for multiple comparisons controlling the family‐wise error (FWE) to be below P_FWE_ < 0.05. Difference in FLAIR hyperintensity (D), cortical thickness (E) and gray–white matter intensity contrast (F) in anterior temporal lobe cluster between TLE patients with (yes) and without (no) a history of febrile convulsions.

**Figure 4:**
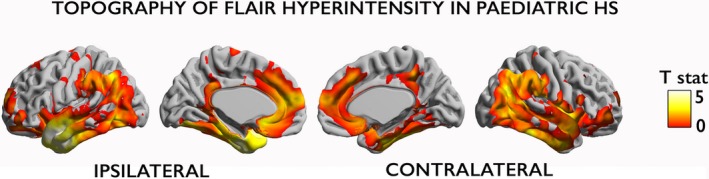
Topography of neocortical FLAIR hyperintensity. Surface‐based map of the *t*‐statistic. Unlike Figure 3A, FLAIR hyperintensity in pediatric TLE patients compared to controls has not been corrected for multiple comparisons.

Compared to controls, patients showed FLAIR signal hyperintensities (Fig. 3A), cortical atrophy (Fig. 3B) and blurring of the gray–white matter boundary (Fig. 3C) in the ipsi‐lesional temporal pole. FLAIR signal hyperintensities and blurring of the gray–white matter boundary were more extensive than cortical thinning. FLAIR hyperintensities involved mesial temporal areas including the parahippocampal gyrus as well as lateral temporal neocortex such as the anterior portion of the superior, middle and inferior temporal gyri. Blurring of the gray–white matter boundary involved the same neocortical areas except there was less involvement of the middle and inferior temporal gyri. In contrast, cortical atrophy only affected a small portion of the temporal pole. Cohen's d effect sizes within temporal pole clusters of findings were large (FLAIR: Cohen's *d* = 1.48; cortical atrophy: *d* = 0.95; gray–white matter blurring: *d* = 2.06). Visualization of the *t*‐statistic of group differences in FLAIR signal intensity (Fig. 4) demonstrated a topographic distribution of FLAIR changes involving bilateral (ipsi > contra‐lateral) temporal and cingulate neocortices as well as the insula. This topography remained the same when repeating the analysis after the exclusion of (*n* = 2) patients with histopathologically confirmed FCD Type IIIa.

### Relation to clinical variables

In patients, MRI morphological and intensity features within the ipsi‐lesional hippocampus were not related to duration of epilepsy, age of seizure onset or history of febrile convulsions. However, in the ipsi‐lateral temporal neocortex, a history of febrile convulsions was associated with increased FLAIR signal intensity (*t* = 1.96, *P* = 0.033 Fig. 3D), when including age of onset and duration of epilepsy as covariates in the regression model. Earlier onset of epilepsy was correlated with more pronounced blurring of the gray–white matter boundary in the ipsi‐lesional temporal pole (*r* = −0.43, *P* = 0.04). Furthermore, patients with temporal lobe blurring reported by the radiologist on their MRI scans had increased quantitatively assessed blurring of the gray–white matter boundary (*t* = 3.06, *P* = 0.004) and FLAIR signal hyperintensity (*t* = 8.31, *P* < 0.001) within the ipsi‐lesional temporal lobe cluster. There was no relationship between MRI morphological or intensity features and duration of epilepsy.

## Discussion

Our quantitative, multimodal framework reveals unilateral hippocampal atrophy and bilateral hippocampal FLAIR hyperintensity in pediatric patients with HS. Hippocampal changes were lateralizing, with more pronounced atrophy and FLAIR signal intensity ipsi‐ versus contra‐lesional. This was as expected given that the inclusion criteria for patients involved a radiological diagnosis of HS. The surface‐based approach, to calculate per‐vertex cortical thickness, normalized FLAIR signal intensity and gray–white matter intensity contrast, demonstrated that while pediatric TLE patients did not show the widespread cortical thinning present in adult cohorts,^16^, ^17^, ^18^, ^19^ they nevertheless had morphological and intensity changes in ipsi‐lesional temporopolar neocortex. Furthermore, temporopolar FLAIR hyperintensities were more marked in patients with a history of febrile convulsions and blurring of the gray–white matter boundary was correlated with earlier onset of epilepsy.

This is the first study to quantify temporopolar abnormalities in children with unilateral HS. The extensive temporopolar FLAIR changes and blurring of the gray–white matter boundary quantified here may be revealing subtle, widespread ipsi‐lesional pathological changes corresponding to the nonspecific histological changes seen in the resected specimens, such as subpial (Chaslin's) gliosis in the gray matter and perivascular glial clustering and perivascular lymphocytes in the white matter. In adults, HS is often observed in conjunction with temporopolar T2/FLAIR signal abnormalities.^33^, ^34^ Our recent study of neocortical changes in adults with TLE showed a paralimbic distribution of FLAIR hyperintensities and indicated a vulnerability of cortex with similar intracortical tissue composition to FLAIR signal hyperintensities. Although, this paralimbic pattern does not survive correction for multiple comparisons in this pediatric cohort; the bilateral paralimbic topographic distribution involving parahippocampal and cingulate cortices as well as the insula is evident (Fig. 4). This supports the idea that these neocortical areas have a shared vulnerability to pathological changes. As FLAIR signal intensity in TLE has been correlated with glial cell count^11^, ^12^, ^13^ and mesiotemporal and neocortical gliosis has been documented in postmortem studies,^2^, ^7^, ^35^ FLAIR changes in the temporal neocortex may indeed reflect gliotic changes. However, T2/FLAIR signal could also reflect alterations in intra‐cortical myelin content,^36^, ^37^ particularly given that a similar distribution of neocortical regions demonstrated quantitative T1 changes in an adult TLE cohort.^38^

Ipsi‐lesional blurring of the gray‐white matter boundary can often be seen on visual inspection of T1w MRI scans of patients with TLE.^39^, ^40^, ^41^ In our study, it was present in 45% of children with HS. This is in line with the literature, which reports that temporopolar blurring is present in 32‐66% of adults with TLE and HS^39^ and in 57% of children.^42^ It has previously been postulated to be caused by vasculometabolic changes,^43^ cortical dysplasia,^44^ inflammatory changes,^45^ increased perivascular spaces, abnormal water content,^46^ widespread gliosis^45^ as well as myelin loss.^45^ A recent study by Garbelli and colleagues relating histopathology to high‐field imaging carefully attempted to disentangle these factors and found that blurring was associated with axonal degeneration and reduced axonal numbers in the white matter.^39^ Interestingly, they observed no difference in blurring between patients with or without subtle cortical dysplasias in the temporal pole. Furthermore, compared to controls, their temporal neocortical specimens did have widespread gliosis and this was unrelated to temporopolar blurring. Thus, blurring of the gray–white matter boundary in children with a radiological diagnosis of HS may likely be driven by axonal changes in the underlying subcortical white matter. However, it is also known that dual pathology can occur in children with HS. Thus, the FLAIR changes and blurring of the gray–white matter boundary found in our cohort of children with HS could be indicative of dual pathology, axonal damage and/or gliotic changes. However, in the present study of children with HS from Great Ormond Street Hospital, when analyses were repeated after removing any patients with histologically proven dual pathology or unclear temporal lobe semiology, the results remained the same. Future work correlating computational anatomy on MRI with histopathology will be required to fully elucidate the underlying etiology of our findings.

Previous studies in adults have documented widespread neocortical atrophy in patients with TLE.^17^, ^18^ Although, pediatric HS patients here showed a cluster of neocortical atrophy in the ipsi‐lesional temporal pole, it is much less extensive than the gray–white matter boundary blurring or FLAIR hyperintensities. This dichotomy between adult and pediatric findings may reflect the fact that pediatric HS patients have shorter durations of epilepsy than adult cohorts. Cortical thinning has previously been associated with duration of epilepsy^18^ and may be related to generalized, seizure‐related factors and reflect consequences of the overall disease process. Importantly however, in this pediatric TLE cohort, there was no correlation with duration of epilepsy. Alternatively it is possible that cortical thinning is a relatively subtle change and requires more statistical power to detect. Moreover, during development, including adolescence, there are widespread changes in cortical tissue composition and connectivity^47^ including in paralimbic networks.^48^ As such, the distributed network of changes seen in adults but not in children might depend on these late‐emerging structural maturations.

Patients with a history of febrile convulsions had more pronounced FLAIR hyperintensity in the ipsi‐lesional temporal pole. This is in accordance with our study in adults with TLE where FLAIR hyperintensity in the parahippocampal gyrus was modulated by a history of febrile convulsions. Furthermore, the results from a prospective imaging study in children have shown visible T2 hyperintensity in the adjacent ipsi‐lesional temporal neocortex following febrile status epilepticus^49^ and suggests a particular vulnerability of the ipsilateral temporal pole to early insults. However, the causal relationship between febrile seizures and the subsequent development of mesial temporal lobe epilepsy is still unclear.^50^

This is the first quantitative evaluation of gray–white matter blurring and FLAIR signal intensity changes in a pediatric TLE cohort. However, HS is a rather uncommon etiology in pediatric epilepsy and as such this study has a modest sample size. Second, as mentioned earlier, it was not possible to disentangle which cytoarchitectural changes (focal cortical dysplasia, gliotic changes, neuronal damage and/or axonal damage) were responsible for the neuroimaging findings reported in this study. Future work combining multimodal neuroimaging with histopathological verification is needed. Incorporating quantitative MR measures such as T1 mapping, which is sensitive to myelin, iron, calcium and free water, and T2 mapping, sensitive to myelin, iron and gliosis, will also assist in this endeavor.^51^

Multimodal structural imaging provides a powerful tool to quantify hippocampal and cortical anomalies in pediatric focal epilepsy. The possibility of extracting quantitative features from multimodal MRI that are sensitive to different aspects of the neuropathology (gliosis, axonal degeneration and demyelination, cortical atrophy) offers the exciting possibility to detect and monitor disease progression and to optimize surgical interventions.

## Author Contributions

SA, JHC, and TB designed the study. SA, SH, BB, NB, AB were involved in the methodological design of the study. SA, MB, GN, RG were involved in data acquisition, review, and analysis. TJ reviewed the histology. JHC reviewed the seizure outcome. SA, MB, GN, RG, SH, BB, NB, AB, TJ, MT, DC, JHC, and TB wrote and revised the paper.

## Conflicts of Interest

No conflicts of interest to declare.
